# Characterization of the Chimeric PriB-SSBc Protein

**DOI:** 10.3390/ijms221910854

**Published:** 2021-10-07

**Authors:** En-Shyh Lin, Yen-Hua Huang, Cheng-Yang Huang

**Affiliations:** 1 Department of Beauty Science, National Taichung University of Science and Technology, No. 193, Sec.1, San-Min Rd., Taichung City 403, Taiwan; eslin7620@gmail.com; 2 School of Biomedical Sciences, Chung Shan Medical University, No. 110, Sec.1, Chien-Kuo N. Rd., Taichung City 402, Taiwan; cicilovev6@gmail.com; 3 Department of Medical Research, Chung Shan Medical University Hospital, No. 110, Sec.1, Chien-Kuo N. Rd., Taichung City 402, Taiwan

**Keywords:** PriB, SSB, PriA, replication fork, PriB-SSBc, DnaT, primosome, OB fold, DNA mimic, GGRQ motif

## Abstract

PriB is a primosomal protein required for the replication fork restart in bacteria. Although PriB shares structural similarity with SSB, they bind ssDNA differently. SSB consists of an N-terminal ssDNA-binding/oligomerization domain (SSBn) and a flexible C-terminal protein–protein interaction domain (SSBc). Apparently, the largest difference in structure between PriB and SSB is the lack of SSBc in PriB. In this study, we produced the chimeric PriB-SSBc protein in which *Klebsiella pneumoniae* PriB (KpPriB) was fused with SSBc of *K. pneumoniae* SSB (KpSSB) to characterize the possible SSBc effects on PriB function. The crystal structure of KpSSB was solved at a resolution of 2.3 Å (PDB entry 7F2N) and revealed a novel 114-GGRQ-117 motif in SSBc that pre-occupies and interacts with the ssDNA-binding sites (Asn14, Lys74, and Gln77) in SSBn. As compared with the ssDNA-binding properties of KpPriB, KpSSB, and PriB-SSBc, we observed that SSBc could significantly enhance the ssDNA-binding affinity of PriB, change the binding behavior, and further stimulate the PriA activity (an initiator protein in the pre-primosomal step of DNA replication), but not the oligomerization state, of PriB. Based on these experimental results, we discuss reasons why the properties of PriB can be retrofitted when fusing with SSBc.

## 1. Introduction

Single-stranded DNA (ssDNA)-binding proteins (SSBs) play crucial roles in DNA replication, repair, recombination, and replication fork restart in both prokaryotes [1] and eukaryotes [2,3,4]. SSB binds to ssDNA with high affinity, regardless of sequence, and prevents premature annealing, chemical attacks, and unwanted nuclease digestion [5]. SSBs typically recognize ssDNA [6,7,8,9] via a highly conserved oligonucleotide/oligosaccharide-binding (OB) fold formed from a five-stranded β-barrel capped by an α-helix [10,11]. The functions of SSB have been studied extensively in *Escherichia coli* (EcSSB) [12,13]. EcSSB consists of an N-terminal ssDNA-binding/oligomerization domain (SSBn) and a flexible C-terminal protein–protein interaction domain (SSBc). SSBc can be further subdivided into two sub-domains, namely, the intrinsically disordered linker (IDL) and the highly conserved acidic tail DDDIPF (SSB-Ct) at the C-terminus. SSB-Ct in SSB can interact with the OB fold and regulate the ssDNA-binding activity of SSB itself [14,15]. Until very recently, IDL, but not just only SSB-Ct, had been found to be involved in binding to at least 20 different partner proteins to regulate the DNA metabolism [1,16,17].

SSB can significantly stimulate the activity of PriA [18], a DEXH-type helicase utilized to reload DnaB back onto the chromosome during replication restart [19,20,21]. DNA fork-bound SSB can load PriA onto the duplex DNA arms of forks [22], and enhance the ability of PriA to discriminate between fork substrates [23]. The ability of PriA-directed replication restart primosome to maintain genetic integrity after encountering DNA damage is essential for bacterial survival [24]. The primosome travels along the lagging strand template, unwinds the duplex DNA, and primes the Okazaki fragments that are required for replication fork progression [25,26]. In addition to PriA, other essential proteins through a series of ordered protein–protein interactions at a repaired DNA replication fork site for a primosome assembly in *E. coli* are PriB, PriC, DnaT, DnaC, DnaB, and DnaG [27]. In a PriA-PriB-DnaT-dependent reaction, PriB is the second protein to participate in the protein–DNA complex [28]. In addition to binding PriB [29,30,31], DnaT is also capable of binding to ssDNA [32,33,34] and PriC [35]. Upon forming the PriA–PriB–DNA complex [36], PriB can induce a conformational alteration in PriA, significantly stimulate the activity of PriA [37], and facilitate the association of DnaT with PriA [38]. PriB can also specifically interact with SSB and ssDNA coated by SSB [39]. Sequence comparisons and operon organization analyses have shown that PriB evolves from SSB [40]. Despite these essential functions, PriB is not absolutely required for bacterial DNA replication [41] and is not present in many bacteria [21,42]. How and why PriB in some bacteria is necessary to evolve from SSB to become a new ssDNA-binding protein during evolution for replication fork restart is still unclear.

**Figure 1:**
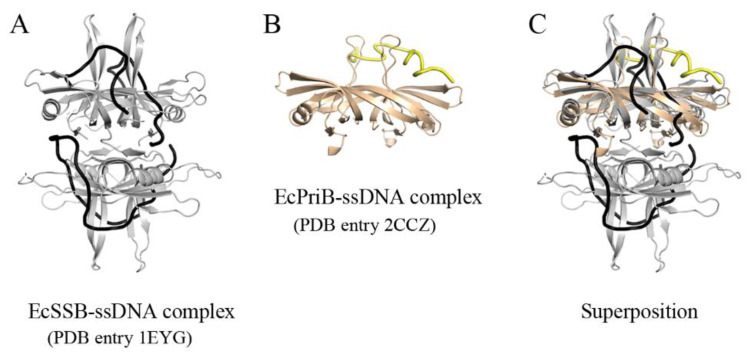
Structural comparison of EcSSB and EcPriB. (**A**) Crystal structure of EcSSB complxed with ssDNA. (**B**) Crystal structure of EcPriB complexed with ssDNA. (**C**) The superimposed structures. The crystal structures reveal that ssDNA wraps around SSB in a binding topology resembling seams on a baseball, while ssDNA adopts an Ω-shaped conformation to bind to the one monomer of the PriB dimer. ssDNAs bound by EcSSB and EcPriB are colored in black and yellow respectively.

PriB presents as a homodimer with two OB folds [43,44,45]. PriB shares structural similarity with its ancestor, SSB; nevertheless, they bind ssDNA differently [46,47]. The crystal structures (Figure 1) reveal that ssDNA wraps around SSB in a binding topology resembling seams on a baseball [9], while ssDNA adopts an Ω-shaped conformation to bind to the one monomer of the PriB dimer [47]. Electrophoretic mobility shift analysis (EMSA) also reveal different ssDNA-binding patterns/behaviors between SSB and PriB [48]. SSB forms multiple distinct complexes with ssDNA of different lengths [49,50,51,52,53], whereas PriB binding to ssDNA of different lengths only forms a single complex [48]. In addition, the ssDNA-binding affinity of PriB is significantly lower (>2–3 orders of magnitude) than that of SSB proteins [48]. The most apparent difference between PriB and SSB is the SSBc; thus, it is worth investigating the effect of SSBc on ssDNA-binding behavior and affinity, the stimulation activity on PriA, and the oligomerization state of PriB.

Chimeragenesis is a powerful technique that creates a protein with improved or new properties to investigate the role of the protein domain(s) by combining different segments originating from different genes [54]. In this study, we produced the chimeric PriB-SSBc protein in which *Klebsiella pneumoniae* PriB (KpPriB) [48] was covalently fused with the SSBc domain of *K. pneumoniae* SSB (KpSSB) [49] to characterize the possible SSBc effects on PriB function. The crystal structure of KpSSB was solved (PDB entry 7F2N) and revealed a novel GGRQ motif in SSBc that pre-occupies and interacts with the ssDNA-binding sites, namely, Asn14, Lys74, and Gln77, in SSBn. Based on these results from biochemical analysis of PriB-SSBc, we found that SSBc could significantly enhance the ssDNA-binding affinity, change the binding behavior, and further stimulate the PriA activity, but not the oligomerization state, of PriB.

## 2. Results

### 2.1. Sequence Analysis between KpPriB and KpSSB

**Figure 2:**
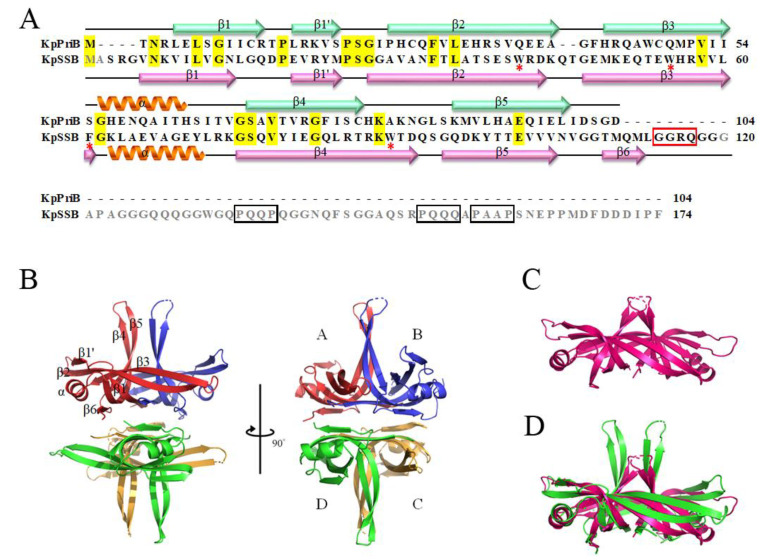
Structural comparison of KpSSB and KpPriB. (**A**) Sequence alignment of KpPriB and KpSSB. Identical residues between KpPriB and KpSSB are shaded in yellow. The corresponding PXXP motifs in KpSSB are boxed in black. The GGRQ motif is boxed in red. The secondary structural elements of KpPriB and KpSSB are shown with the sequences. The amino acids 120–174 (in gray) in the structure of KpSSB were not observed. (**B**) Crystal structure of KpSSB (PDB entry 7F2N). Four monomers of KpSSB were found per asymmetric unit. The KpSSB monomer has an OB-fold domain similar to EcSSB, and the core of the OB-fold domain possesses a β-barrel capped with an α-helix. The amino acids 120–174 in the structure of KpSSB were not observed. (**C**) Crystal structure of KpPriB (PDB entry 4APV). (**D**) The superimposed structures of KpPriB and the KpSSB dimer. The KpSSBn and KpPriB are similar, in which the only significant difference is in the lengths of the β4 and β5 sheets.

PriB shares structural similarity with the ssDNA-binding domain of SSB [43,44,45]. Given the structural resemblance, one may conclude that PriB binds ssDNA in a manner similar to SSB. However, the complex structure reveals that PriB binds ssDNA differently [47]. Despite the similar architecture, the amino acid sequences of PriB and SSB from K. pneumoniae share only 11% identity and 29% similarity (Figure 2A). In the EcSSB-ssDNA complex [9], four essential aromatic residues, Trp40, Trp54, Phe60, and Trp88, participate in ssDNA binding via stacking interactions. These residues (marked by asterisks) are conserved in KpSSB but not in KpPriB (Figure 2A). Unlike SSB, PriB does not possess IDL and SSB-Ct. The PXXP motifs in the IDL of EcSSB are known to mediate the protein–protein interactions [17,55]. In EcSSB, the PXXP motifs occur at residues 139 (PQQP), 156 (PQQS), and 161 (PAAP). The corresponding motifs in KpSSB (boxed in black) are PQQP, PQQQ, and PAAP, respectively (Figure 2A). The crystal structure of KpSSB (Figure 2B) solved in this study revealed a novel GGRQ motif (boxed in red), which might be involved in regulating the ssDNA binding (see below).

### 2.2. Protein Chimeragenesis

KpPriB (Figure 2C) and KpSSB (Figure 2D) are OB-fold proteins with different ssDNA binding behaviors [48,49]. However, sequence comparisons and operon organization analyses indicate that PriB evolved from SSB via gene duplication with subsequent rapid sequence diversification [40]. The significant difference between PriB and SSB is the protein length; that is, PriB does not have SSBc consisting of IDL and SSB-Ct. IDL [56] and SSB-Ct [18,57,58,59] in SSB are required to stimulate the activity of PriA. Interestingly, PriB does not possess SSBc but can still stimulate the activity of PriA [37]. Thus, we attempted to obtain and characterize the chimeric protein PriB-SSBc in which KpPriB were fused with KpSSBc at the C termini of KpPriB. PriB-SSBc possessing both characteristics of PriB and SSBc was then used to analyze whether the SSBc can change the properties of PriB, such as the oligomeric state, the ssDNA-binding behavior, and the stimulating effect on PriA, in situ.

**Figure 3:**
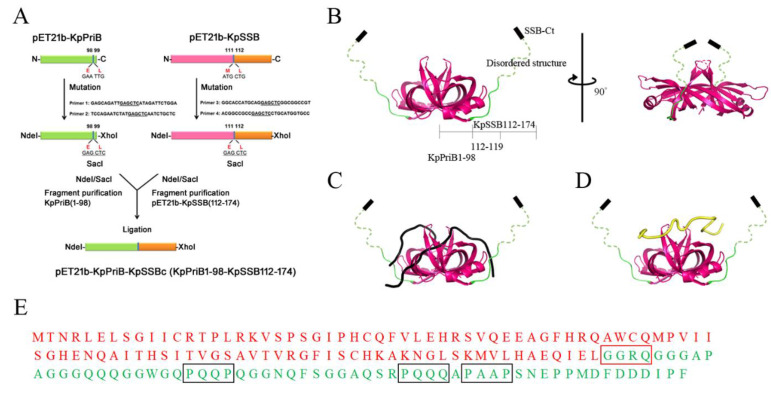
The chimeric PriB-SSBc protein. (**A**) Construction of the plasmid for expression of the chimeric PriB-SSBc protein. The resultant plasmid pET21b-KpPriB-KpSSBc will express KpPriB1–98 fused KpSSB112–174, designated as PriB-SSBc. Note that PriB-SSBc has 161 amino acid residues and does not have any artificial residues. (**B**) A proposed structure of PriB-SSBc. The structure was directly constructed by superimposing the KpPriB dimeric form (aa 1–98; PDB entry 4APV) with the crystal structure of KpSSB (aa 112–119; PDB entry 7F2N). The unobserved region (aa 120–174) in KpSSBc was shown as dashed lines. The ssDNA-binding property of PriB-SSBc may resemble that of (**C**) EcSSB or (**D**) EcPriB. (**E**) The putative amino acid sequence of PriB-SSBc.

We constructed the plasmid to express the chimeric protein PriB-SSBc following several steps (Figure 3A). To obtain an additional cutting site (SacI) for fusing PriB and SSBc fragments, the pET21b-PriB [48] and pET21b-KpSSB (with the stop codon to avoid having a His tag fused with the gene product) [60] plasmids were mutated to create a desired SacI restriction site (aa 98–99 for pET21b-PriB/SacI and aa 111–112 for pET21b-SacI/KpSSB, respectively). The pET21b-PriB/SacI plasmid was cut with NdeI and SacI restriction enzymes, and the fragment KpPriB(1–98) was purified. Meanwhile, the pET21b-SacI/KpSSB plasmid was also treated with NdeI and SacI restriction enzymes, and the resultant DNA fragment pET21b-KpSSB(112–174) was purified and then ligated with the insert KpPriB(1–98) DNA fragment. The resultant plasmid pET21b-KpPriB-KpSSBc will express KpPriB1–98 fused KpSSB112–174 (Figure 3B), designated as PriB-SSBc in this study. Given that PriB-SSBc inherited a DNA-binding domain from KpPriB, this chimeric protein might be thought to bind ssDNA in a manner similar to that of KpPriB, not KpSSB. To confirm this, we analyzed whether the ssDNA-binding property of PriB-SSBc resembles that of SSB (Figure 3C) or PriB (Figure 3D). Note that PriB-SSBc has 161 amino acid residues and does not have any artificial residues (Figure 3E).

### 2.3. Purification of KpPriB, KpSSB, and PriB-SSBc

**Figure 4:**
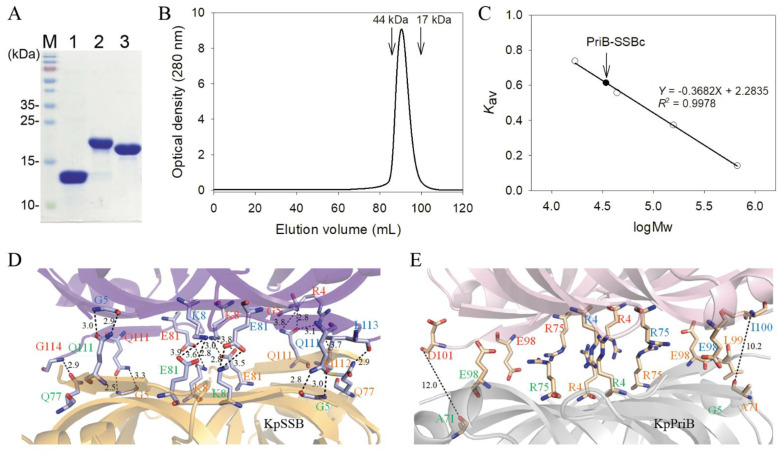
Oligomeric state of PriB-SSBc. (**A**) Protein purity. Coomassie Blue-stained SDS-PAGE (15%) of the purified KpPriB (lane 1), KpSSB (lane 2), PriB-SSBc (lane 3), and molecular mass standards are shown. (**B**) Gel-filtration chromatographic analysis of the purified PriB-SSBc. The corresponding single peak shows the eluting PriB-SSBc. (**C**) Native molecular mass of PriB-SSBc. The native molecular mass of PriB-SSBc was estimated to be 34209 Da, approximately twice as large as that of a PriB-SSBc monomer. (**D**) Structural analysis of the dimer–dimer interface of KpSSB. Many hydrogen bonds and salt bridges were formed at the dimer–dimer interface of KpSSB. These residues from the subunit A, B, C, and D are labeled in red, blue, orange, and green, respectively. The distance (Å) of the residues is also shown. (**E**) The superposition of two PriB dimers as the architecture of the KpSSB tetramer. The corresponding residues of KpPriB are not conserved and too far away to interact with each other. Some of them are near to one another but exhibit charge repulsion.

KpPriB, KpSSB, and PriB-SSBc were hetero-overexpressed in *E. coli*. These gene products did not have a His tag to avoid any artificial effects for further analysis. KpSSB was purified by the precipitation of ammonia sulfate, Q, and Heparin column chromatographies (Figure 4A). Unlike KpSSB, recombinant KpPriB and PriB-SSBc could be purified from the soluble supernatant only in a single chromatographic step using the SP column by the AKTA-FPLC system (Figure 4A).

### 2.4. Oligomeric State of PriB-SSBc in Solution

PriB and SSB form dimers and tetramers, respectively [45]. Whether or not PriB-SSBc can form dimers, tetramers, or a mixture of dimers and tetramers remains to be elucidated. The analysis of purified PriB-SSBc (4 mg/mL) by gel filtration chromatography showed a single peak with an elution volume of 90.5 mL (Figure 4B). Assuming that PriB-SSBc has a shape and partial specific volume similar to the standard proteins, the native molecular mass of PriB-SSBc was estimated to be 34209 Da, calculated from a standard linear regression equation, K_av_ = −0.3682 (logMw) + 2.2835 (Figure 4C). The native molecular mass for PriB-SSBc is approximately twice as large as that of a PriB-SSBc monomer (approximately 17.8 kDa). Accordingly, we concluded that PriB-SSBc in solution is a stable dimer like KpPriB [48], but not a tetramer like KpSSB [49]. The order of the native molecular size was as follows: KpSSB > PriB-SSBc > KpPriB.

### 2.5. Crystal Structure of KpSSB

The crystallization of PriB-SSBc was attempted, but we could not obtain any crystal to solve the crystal structure after the initial screening. The structure of KpPriB was available (PDB entry 4APV); thus, we attempted to obtain the crystal structure of KpSSB and combined their structural features to visualize PriB-SSBc. We crystallized KpSSB through hanging drop vapor diffusion and determined its structure at a resolution of 2.35 Å (Table 1). The crystal of KpSSB belonged to space group C_1_2_1_ with cell dimensions of a = 108.39, b = 57.01, and c = 93.80 Å. Four monomers of KpSSB per asymmetric unit were present (Figure 2B). Accordingly, KpSSB forms a tetramer in solution [49].

The secondary structural element of KpSSB is similar to that of KpPriB (Figure 2A), but significant differences in the lengths of β4- and β5-sheets were found (Figure 2C,D). The KpSSB monomer has an OB-fold domain similar to EcSSB, and the core of the OB-fold domain possesses a β-barrel capped with an α-helix. Unlike *Streptomyces coelicolor* SsbB [61], *Staphylococcus aureus* SsbA (SaSsbA) [60] and SaSsbB [58,62], KpSSB contained additional β6 strand. β6 strands function by clamping two neighboring subunits together in a tetrameric SSB [61]. Thus, KpSSB may exhibit different protein–DNA and protein–protein interaction specificities from these Gram-positive bacterial SSBs. The amino acids 120–174 in the structure of KpSSB were not observed, suggesting that the C-terminal region in KpSSB was dynamic, similar to that in EcSSB [63]. As compared to the crystal structure of the full-length EcSSB (PDB entry 1SRU) [63], six additional residues (amino acids 114–119; GGRQGG) were determined in KpSSB.

The structures of KpSSB (Figure 4D) and KpPriB (Figure 4E) were used to explain why PriB-SSBc could not form a tetramer. Many hydrogen bonds and salt bridges were formed at the dimer–dimer interface of KpSSB (Table 2). The superposition of two PriB dimers as the architecture of the KpSSB tetramer revealed that the corresponding residues of KpPriB are not conserved and too far away to interact with each other. Some of them are near to one another but exhibit charge repulsion (e.g., R4–R75). Thus, KpPriB and PriB-SSBc could not form a tetramer as with KpSSB.

### 2.6. Binding of PriB-SSBc to ssDNA

**Figure 5:**
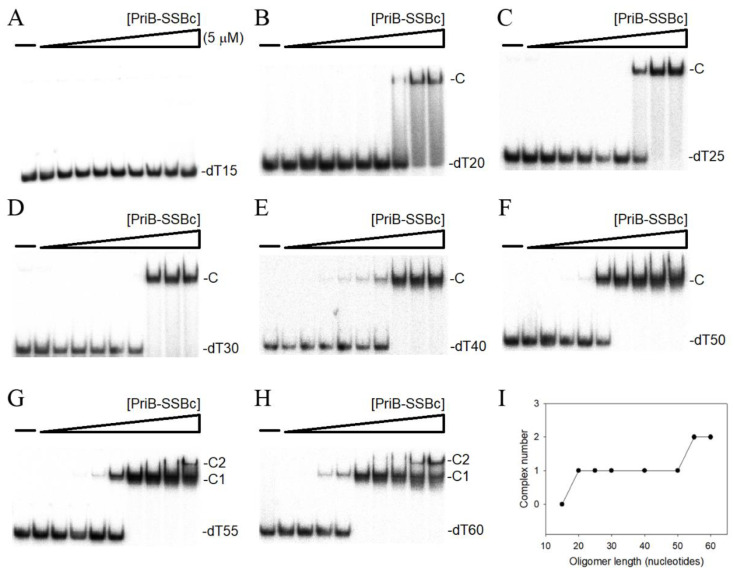
EMSA of PriB-SSBc. Protein (0, 19, 37, 77, 155, 310, 630, 1250, 2500, and 5000 nM) was incubated at 25 °C for 30 min with 1.7 nM of (**A**) dT15, (**B**) dT20, (**C**) dT25, (**D**) dT30, (**E**) dT40, (**F**) dT50, (**G**) dT55, or (**H**) dT60 in a total volume of 10 μL in 20 mM Tris–HCl (pH 8.0) and 100 mM NaCl. (**I**) Summary of the complex number of PriB-SSBc. Complex number of PriB-SSBc as a function of the length of the ssDNA determined using EMSA.

SSB-Ct in SSB can interact with the OB fold and regulate the ssDNA-binding activity [14,15]. Expectedly, it might also mean that the SSBc (including IDL and SSB-Ct) in PriB-SSBc is capable of interacting with the ssDNA-binding sites within the OB fold and inhibit the ssDNA binding of PriB-SSBc itself. Thus, we attempted to test whether or not PriB-SSBc has ssDNA-binding activity. If so, we are also interested in whether PriB-SSBc has a lower binding ability than that of PriB; that is, the presence of SSBc in PriB-SSBc may physically or hinderingly inhibit the binding process. We studied the binding of PriB-SSBc to ssDNA of different lengths with different protein concentrations using the electrophoretic mobility shift analysis (EMSA). EMSA is a well-established approach in studies of molecular biology, allowing the detection of the distinct protein–DNA complex(es) [64]. The expected result of EMSA is that when the length of the nucleotides is sufficient for the binding of two or more protein molecules, the electrophoretic mobility of the higher SSB oligomer complex will be lower than that of the smaller protein oligomer complex. When we incubated PriB-SSBc with a 15-mer deoxythymidine oligonucleotide (dT15), no band shift was observed, indicating that PriB-SSBc could not form a stable complex with this homopolymer (Figure 5A). We further tried to use longer ssDNA homopolymers for the binding of PriB-SSBc. In contrast to dT15, longer dT homopolymers, dT20–50 (Figure 5B–F), produced a very significant band shift (C, complex). These findings confirm the ssDNA-binding activity of PriB-SSBc, which is strong enough to form a stable protein–DNA complex in solution. Furthermore, two different complexes for dT55 and dT60 were formed by PriB-SSBc (Figure 5G,H). At lower protein concentrations, PriB-SSBc formed a single complex (C1) with dT55, similar to that observed with dT50 (Figure 5F). However, when the PriB-SSBc concentration was increased, another slower-migrating complex (C2) was observed (Figure 5G). The appearance of the second complex resulted from the increased PriB-SSBc concentration, suggesting that two PriB-SSBc molecules may be contained per oligonucleotide (Figure 5I). Although dT55 is only 5 nt longer than dT50, the presence of an extra 5 nt in dT55 compared with that of dT50 provides enough interaction space for the binding of two PriB-SSBc dimers. Therefore, one PriB-SSBc occupies 25 (50/2 = 25) to 27.5 (55/2 = 27.5) nt of the ssDNA. These results from EMSA suggest that the length of an ssDNA required for PriB-SSBc binding is 26 ± 2 nt.

### 2.7. Binding of KpPriB to ssDNA

**Figure 6:**
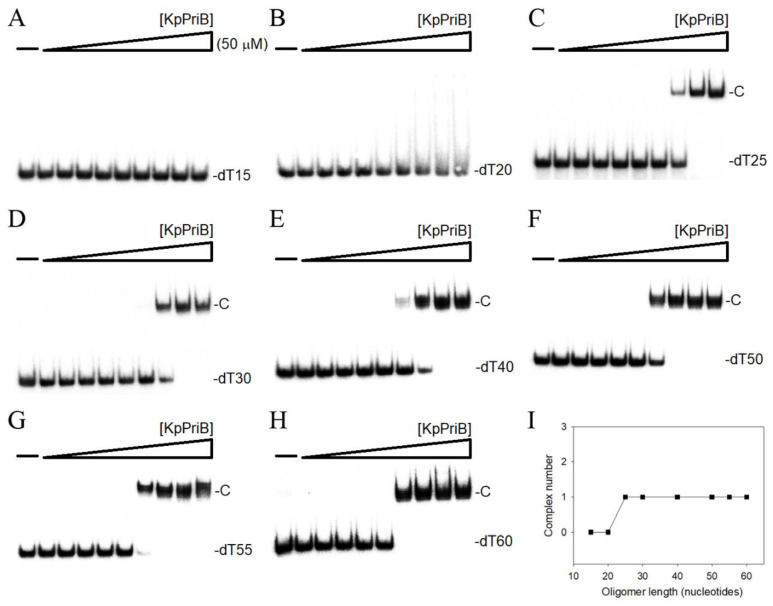
EMSA of KpPriB. Protein (0–50 μM) was incubated at 25 °C for 30 min with 1.7 nM of (**A**) dT15, (**B**) dT20, (**C**) dT25, (**D**) dT30, (**E**) dT40, (**F**) dT50, (**G**) dT55, or (**H**) dT60 in a total volume of 10 μL in 20 mM Tris–HCl (pH 8.0) and 100 mM NaCl. (**I**) Summary of the complex number of KpPriB. Complex number of KpPriB as a function of the length of the ssDNA determined using EMSA.

In order to compare KpPriB to PriB-SSBc, its binding to ssDNA of different lengths was studied. An EMSA of the binding of KpPriB to dT15–dT60 with different protein concentrations was performed. KpPriB could not form a stable complex with dT15 (Figure 6A). Unlike PriB-SSBc, KpPriB could not form a stable complex with dT20 (Figure 6B). As some smears were observed, it appears that KpPriB can interact with dT20. In contrast to dT20, the longer dT homopolymers (dT25–dT60) can bind to KpPriB and form a single complex (Figure 6C–I). Unlike the case of PriB-SSBc, no other obvious complex was detected for the binding of KpPriB to dT55 and dT60. These interactions appear to be highly cooperative as only one complex of KpPriB molecules bound per ssDNA was visible. Accordingly, we concluded that KpPriB bound ssDNA differently to that of PriB-SSBc.

### 2.8. Binding of KpSSB to ssDNA

**Figure 7:**
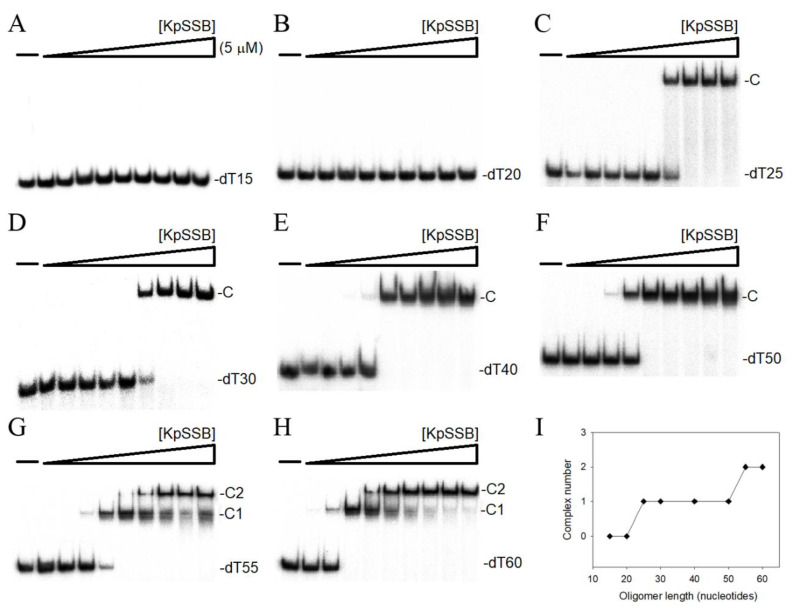
EMSA of KpSSB. Protein (0–5 μM) was incubated at 25 °C for 30 min with 1.7 nM of (**A**) dT15, (**B**) dT20, (**C**) dT25, (**D**) dT30, (**E**) dT40, (**F**) dT50, (**G**) dT55, or (**H**) dT60 in a total volume of 10 μL in 20 mM Tris–HCl (pH 8.0) and 100 mM NaCl. (**I**) Summary of the complex number of KpSSB. Complex number of KpSSB as a function of the length of the ssDNA determined using EMSA.

The binding of KpSSB to ssDNA of different lengths (dT15–60) was also analyzed (Figure 7). KpSSB could not form a stable complex with dT15 or dT20 (Figure 7A,B). Except for binding to dT20 (Figure 5B), binding patterns of KpSSB to other dT homopolymers were similar to those of PriB-SSBc (Figure 7C–I). Similar to PriB-SSBc (Figure 5G), two different complexes with dT55 were observed for higher concentrations of KpSSB (Figure 7G), suggesting the binding of two KpSSB tetramers on a single ssDNA. As one KpSSB occupies 25 (50/2 = 25) to 27.5 (55/2 = 27.5) nt of the ssDNA, the length of an ssDNA required for KpSSB binding is 26 ± 2 nt. Interestingly, KpSSB has more OB-fold domains than PriB-SSBc and is thought to have more ssDNA-contacting sites; however, KpSSB did not bind the shorter ssDNA with dT20 effectively, but PriB-SSBc did. KpPriB also did not bind to dT20. The reason that additional SSBc linked with KpPriB can enhance the ssDNA-binding activity (Table 3) and change the binding behavior from KpPriB to KpSSB remains unclear. A co-crystal structure of PriB-SSBc is needed to compare their ssDNA-binding modes.

### 2.9. Binding Constants of the SSB–ssDNA Complexes Determined from EMSA

To compare the ssDNA-binding abilities of PriB-SSBc, KpPriB, and KpSSB, the midpoint values for input ssDNA binding, calculated from the titration curves of EMSA and referred to as [Protein]_50_ (monomer), were quantified and are summarized in Table 3. Although these proteins possess similar ssDNA-binding domains, their ssDNA-binding activities and complex-forming patterns are different (Table 3). [PriB-SSBc]_50_ values ranged from 0.23 to 2.12 μM; [KpPriB]_50_ values ranged from 4.8 to 14.1 μM; and [KpSSB]_50_ values ranged from 0.05 to 0.58 μM. The ssDNA-binding ability is as follows: KpSSB > PriB-SSBc > KpPriB. Results from the above analyses indicated that SSBc fused with PriB significantly changed the ssDNA-binding properties, including the increase in the binding ability and the formation of distinct complexes.

### 2.10. PriB-SSBc Could Significantly Stimulate the ATPase Activity of KpPriA

**Figure 8:**
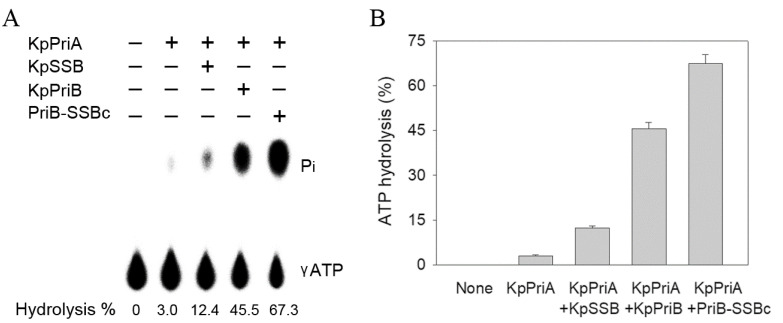
ATPase activity of KpPriA. (**A**) KpPriA ATPase assay was performed with 0.4 mM [γ^−32^P] ATP and 0.025 μM KpPriA in reaction buffer containing 40 mM Tris (pH 8.0), 10 mM NaCl, 2 mM DTT, 2.5 mM MgCl_2_, and 0.1 μM PS4/PS3-dT30 DNA substrate. To study the effect, KpSSB (10 μM), KpPriB (10 μM), or PriB-SSBc (10 μM) was added into the assay solution. Aliquots (5 μL) were taken and spotted onto a polyethyleneimine cellulose thin-layer chromatography plate, which was subsequently developed in 0.5 M formic acid and 0.25 M LiCl for 30 m. Reaction products were visualized by autoradiography and quantified with a phosphorimager. (**B**) The stimulating effect on KpPriA. The ATPase activity of KpPriA stimulated by KpSSB, KpPriB, and PriB-SSBc was enhanced by 4-, 15-, and 22-fold, respectively.

PriB [37] and SSB [18], but not KpSSBc [59], can significantly stimulate the activity of PriA. To investigate whether or not PriB-SSBc can stimulate the activity of KpPriA as KpSSB does [56,59], the ATPase activity of KpPriA was assayed in the presence of PriB-SSBc (Figure 8A). KpPriB was also used for comparison (Figure 8A). KpPriA could hydrolyze ATP alone, and this ATPase activity was dramatically stimulated when KpSSB, KpPriB, and PriB-SSBc were individually present (Figure 8A). The ATPase activity of KpPriA stimulated by KpSSB, KpPriB, and PriB-SSBc was enhanced by 4-, 15-, and 22-fold, respectively. The stimulating effect on the activity of KpPriA was as follows: PriB-SSBc > KpPriB > KpSSB. The enhancing ability for PriB-SSBc was significantly greater than that of KpPriB and KpSSB (Figure 8B). Thus, KpPriB acting with SSBc (PriB-SSBc) had a synergistic effect on PriA stimulation.

### 2.11. The 114-GGRQ-117 Motif as a Regulatory Switch for ssDNA Binding

**Figure 9:**
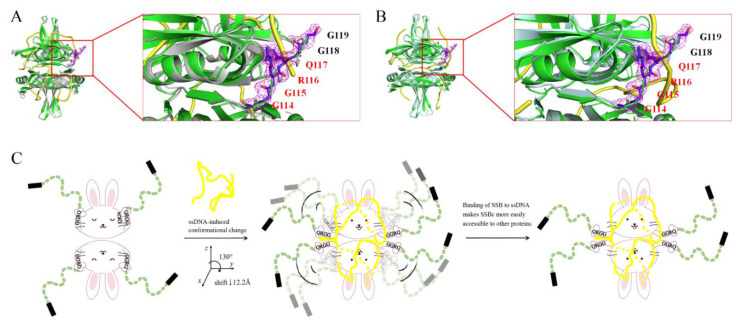
The 114-GGRQ-117 motif as a regulatory switch for ssDNA binding. (**A**) The superimposed structures of KpSSB and the EcSSB-ssDNA complex. KpSSB and EcSSB are colored in green and gray, respectively. ssDNA is colored in yellow. (**B**) The superimposed structures of KpSSB and the PaSSB-ssDNA complex. PaSSB is colored in pale cyan. Six additional residues, 114-GGRQGG-119, were observed in the structure of KpSSB. This region was not visible in the structures of the EcSSB- and PaSSB–ssDNA complexes. The 114-GGRQ-117 motif labeled in red interacted with Asn14, Lys74, and Gln77 in KpSSBn via several hydrogen bonds. The GGRQ motif-interacting residues Asn14, Lys74, and Gln77 in KpSSB are ssDNA-binding residues in EcSSB- and PaSSB–ssDNA complexes. (**C**) A cartoon model. The GGRQ motif might be a regulatory switch for SSBn or ssDNA binding. In the structure of apo-KpSSB, the GGRQ motif interacted with Asn14, Lys74, and Gln77 via several hydrogen bonds. Superimposing analysis indicated that if binding of the EcSSB–ssDNA complex occurs, the GGRQ peptide in KpSSB will break free from these hydrogen bonds and shift away by a distance of 12.2 Å and angles of 130˚ to form a complex with ssDNA. The binding of ssDNA might be a driving force to promote the conformational change in the GGRQ motif. Thus, binding of SSB to ssDNA makes SSBc more easily accessible to other proteins.

IDL in SSB can bind to the OB fold in the absence of ssDNA [65]. When SSBn binds to ssDNA, IDL is no longer bound by SSBn [65]. Thus, we checked whether any residues originally defined for ssDNA binding also interacted with IDL as seen in our KpSSB structure. As compared with the crystal structures of the EcSSB- (Figure 9A) and *Pseudomonas aeruginosa* SSB (PaSSB)–ssDNA complex (Figure 9B), we found that a GGRQ motif occurs at residues 114, which might be a regulatory switch for SSBn or ssDNA binding. In the structure of KpSSB, the GGRQ motif interacted with Asn14, Lys74, and Gln77 via several hydrogen bonds (Table 4). These GGRQ motif-interacting residues (Asn14, Lys74, and Gln77) in KpSSB, perfectly conserved in EcSSB and PaSSB [49], are ssDNA-binding residues in EcSSB- and PaSSB–ssDNA complexes [7,9]. In the structure of the PaSSB–ssDNA complex, the GGRQ motif is not observed probably due to disorder [7]. Superimposing analysis indicated that if binding of the EcSSB–ssDNA complex occurs, the GGRQ peptide in KpSSB will break free from several hydrogen bonds (Table 4) and shift away by a distance of 12.2 Å and angles of 130˚ to form a complex with ssDNA (Figure 9C). The binding of ssDNA might be a driving force to promote the conformational change in the GGRQ motif (Figure 9C). Our structural evidence supports the role of the GGRQ motif as a regulatory switch via the conformational change of binding ssDNA to SSB. Given that the position of the GGRQ motif is located at the ssDNA binding path of SSB, whether or not this motif is also involved in altering the SSB_35_/SSB_65_ distribution and causes different SSB binding modes should be further elucidated.

### 2.12. Analysis of the Ssb and PriB Genes

**Figure 10:**
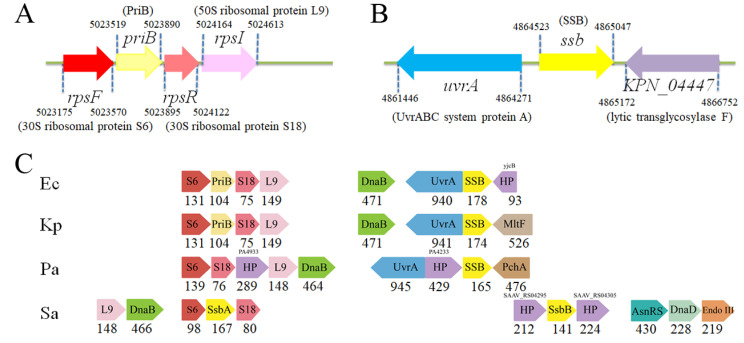
Gene map of *K. pneumonia* chromosomal region with *priB* and *ssb*. (**A**) The gene coding for KpPriB maps from the 5023519 to 5023890 nt of the *K. pneumonia* genome. This *priB* gene is flanked by the *rpsF* and *rpsR* genes, coding for the ribosomal proteins S6 and S18, respectively. (**B**) The gene coding for KpSSB maps from the 4864523 to 4865047 nt of the *K. pneumonia* genome. This *ssb* gene is located adjacent to the *uvrA* and *KPN_04447* genes, coding for the excinuclease ABC subunit UvrA and lytic transglycosylase F (also identified as the periplasmic binding protein MltF), respectively. (**C**) Gene arrangements around the *priB* and *ssb* genes in different bacterial genomes. The *priB* gene organization in *K. pneumonia*, as well as in the Gram-negative bacterium *E. coli* (but not *P. aeruginosa*), resembles *ssb* (*ssbA*; the main *ssb*) gene organization in the Gram-positive bacteria *S. aureus* and *B. subtilis*. Gene maps of the DnaB helicase, DnaD loader, and SsbB (the second *ssb*) are also shown. The number is the gene product length (aa). HP, hypothetical protein; L9, the 50S ribosomal protein L9; PchA, isochorismate synthase; AsnRS, asparaginyl-tRNA synthetase.

We analyzed the *priB* (*KPN_04595*) (Figure 10A) and *ssb* (*KPN_04446*) (Figure 10B) gene maps from *K. pneumonia* using a database search through the National Center for Biotechnology Information. The *priB* gene is flanked by the *rpsF* and *rpsR* genes, coding for the ribosomal proteins S6 and S18, respectively. Interestingly, this *priB* gene organization in *K. pneumonia* (Figure 10C), as well as in the Gram-negative bacterium *E. coli* (but not *P. aeruginosa*), resembles *ssb* (*ssbA*; the main *ssb*) gene organization in the Gram-positive bacteria *S. aureus* (Figure 10C) and *B. subtilis*. The *ssb* gene coding for *Deinococcus radiodurans* SSB, a homodimeric SSB in which each monomer contains two OB folds [66], is also embedded within a ribosomal protein operon (data not shown). Given that these genes (*rpsF*, *ssbA*, and *rpsR*) in Gram-positive *B. subtilis* belong to one operon and are controlled by the SOS response [67], the *priB* gene might be also controlled by the SOS response in the Gram-negative bacteria. This result from the gene map analysis may explain why it is not necessary to synchronically express PriB with PriA and DnaT even at elevated pressure when *E. coli* growth occurs [68]. Given that many prokaryotic genomes do not contain a recognizable homolog of *priB* and *dnaT* (e.g., *P. aeruginosa*) [21], further operon and gene regulation analyses for PriB and DnaT expression, not limited to replication restart, should also be investigated in combination with biochemical and structural investigations.

Unlike *ssbA* (*S. aureus*) and *priB* (*K. pneumonia* and *E. coli*) embedded within a ribosomal protein operon, *E. coli* and *K. pneumonia ssb* genes are located adjacent to the *uvrA* gene (Figure 10C). For physiological needs, these genes coding for SSB proteins would, therefore, be gradually different in structure and function under different regulation signaling pathways during evolution.

## 3. Discussion

It is believed that all cells present now evolved from a common ancestor, implying that the basic principles learned from experiments performed with one type of cell should be generally applicable to other cells. Accordingly, mechanisms of many fundamental cellular activities, such as DNA replication, transcription, and translation, in different types of cells should be similar. Due to stressful environmental conditions, however, organisms will evolve new enzymes or auxiliaries for better survival and to increase their adaptability during evolution. For example, the three eukaryotic nuclear RNA polymerases carry out transcription to copy a segment of DNA into RNA, and they all seem to have evolved from a single enzyme present in the common ancestor with archaea [69]. However, the evolution of SSB may be not the case for the polymerase. Although SSBs from eubacteria [12] to higher eukaryotes (e.g., RPA) [3] share basic mechanistic functioning, such as ssDNA binding and protection from damage during DNA replication, they are different in terms of structure and many other functions [56,59,63,70,71,72]. In addition, many bacteria have more than one paralogous SSB, such as SsbA [59], SsbB [58,62], and SsbC [57], in *S. aureus*. In *E. coli*, PriB is also identified as a kind of SSB [45,47]. Thus, the presence of these diverse SSBs may indicate that SSB must co-evolve with the partner proteins to develop a unique function in each species according to survival needs and obtain a competitive edge. For example, the amino acid residues of IDL in different SSBs are not conserved [56]. Further research is still needed to clarify why it is necessary to evolve these different SSBs in particular species and whether and how their physiological functions are different from the main SSB.

Although PriB is essential in the pre-primosomal step of DNA replication [73], PriB is only found in β- and some γ-proteobacteria [40]. In these bacteria, the *priB* gene is embedded within a ribosomal protein operon [74]. Many ribosomal proteins which possess OB-fold domains are RNA-binding proteins [75,76]. As prokaryotic operons typically encode functionally linked proteins [77,78], PriB might also function as an RNA-binding protein and may be involved in RNA metabolism together with the ribosomal proteins S6 and S18 (Figure 10). Indeed, unlike SSB, which prefers to bind to ssDNA [5], PriB binds to ssDNA and RNA with comparable affinity [45]. Thus, the possibility that PriB plays a role in physiology functioning to bind RNA (e.g., RNA chaperone [79,80,81,82]) still cannot be ruled out at this time. However, this speculation must be further genetically and structurally elucidated.

The *priB*-coded site (Figure 10) in the operon is replaced by the main *ssb* gene in ε-proteobacteria and many other bacteria [40]. The respective main *ssb* genes in the Gram-positive and -negative bacteria are located far apart and embedded within different operons. It appears reasonable that the duplication of the *ssb* gene was accompanied by a genome rearrangement, which resulted in one of the paralogs retaining the original position, whereas the other one was relocated [40]. Interestingly, the original SSB function remained with the relocated paralog, whereas the one within the ribosomal protein operon acquired a new function, such as PriB, a component for the PriA-directed primosome assembly. For PriB-lacking bacteria (e.g., the Gram-positive *S. aureus* and *B. subtilis*), some auxiliary proteins, such as DnaD [82,83], would, therefore, evolve for the need of the PriA-directed primosome assembly (Figure 10C). Interestingly, the Gram-negative *P. aeruginosa* does not contain any recognizable homolog of *priB*, *dnaT*, *priC*, and *dnaC* in its genome [56]. For the restart system, only PriA and SSB are found in *P. aeruginosa*. Whether the PriA-directed primosome in *P. aeruginosa* exists and how it recalls the DnaB helicase back onto the chromosome is yet to be elucidated.

Unexpectedly, the ssDNA-binding affinity of PriB-SSBc is significantly higher than that of KpPriB (Table 3). SSBc cannot bind to ssDNA but is capable of enhancing the KpPriB’s binding affinity to ssDNA when covalent fusion occurs. Whether SSBc can facilitate the recognition of KpPriB to ssDNA or increase the contact region of KpPriB to ssDNA remains to be demonstrated. To further elucidate how the SSBc can improve the activity of KpPriB, the crystal structure of PriB-SSBc in complex with ssDNA is highly desired.

Many SSB proteins bind to ssDNA with some degree of positive cooperativity [84]. In this study, we found different EMSA behaviors among KpPriB, PriB-SSBc, and SSB proteins (Figure 5, Figure 6 and Figure 7). SSB proteins form multiple distinct complexes with ssDNA of different lengths [50,51,53], whereas KpPriB binding to ssDNA of different lengths only forms a single complex [48]. When fusing with SSBc, the EMSA behavior of KpPriB was almost shifted to that of KpSSB. EMSA with the use of a radioactive tracer is a useful technology in molecular biology [85], allowing the detection of the distinct protein–DNA complex(es) [64]. The ssDNA binding patterns of PriB-SSBc did not resemble those of KpPriB; thus, SSBc plays a significant role in regulating the binding mode. These findings also raise several questions as to why PriB has become a new kind of SSB but compensates for the loss of SSBc. Apparently, the loss of SSBc leads to a decrease in the ssDNA-binding ability of KpPriB as compared with that of KpSSB, KpPriB, and PriB-SSBc. Why does PriB participate in DNA replication differently from its ancestor (SSB)? If tolerable, the PriB progenitor will not abandon SSBc, an essential protein fragment for many cellular uses [1,16,17,55,86,87,88].

We demonstrated that the stimulating effect on the activity of KpPriA was as follows: PriB-SSBc > KpPriB > KpSSB (Figure 8). It is known that PriB stimulates PriA via an interaction with ssDNA [37]. Given that the ssDNA-binding ability of KpPriB was lower than that of KpSSB (Table 3), why KpPriB can stimulate the activity of PriA more than that induced by KpSSB is unclear (Figure 8). Whether it was caused due to PriB using a different ssDNA-binding strategy to SSB remains unclear [47]. SSBn [59] and SSBΔC10 [18] cannot stimulate PriA. Thus, the specific protein–protein interactions, such as within DnaD [83], are also important for the stimulation effect on PriA. For PriB-SSBc, it is easy to tentatively speculate that the highest stimulation activity results from the co-action of PriB with SSBc. The synergistic effect may mean that PriA has more than one access site for stimulation.

To date, the structure of SSBc has not been observed. As compared to EcSSB, the crystal structure of KpSSB solved in this study at a resolution of 2.3 Å (Table 1) revealed the structure of the six additional residues 114-GGRQGG-119 (Figure 9) in SSBc. Based on this structure, we identified the GGRQ motif as a regulatory switch for controlling the binding of either SSBn or ssDNA. IDL in SSB can bind to SSBn in the absence of ssDNA [65]; when the binding of SSBn to ssDNA occurs, IDL is no longer bound by SSBn [65]. Here, our structure provided convincing evidence for this hypothesis. The GGRQ motif forms several hydrogen bonds with Asn14, Lys74, and Gln77 in KpSSB (Table 4). Correspondingly, Asn14, Lys74, and Gln77 in EcSSB [9] and PaSSB [7] are ssDNA-interaction sites revealed by the complex structures. Thus, we propose that a cycle of conformational changes in GGRQ with SSBn is associated with ssDNA binding. When binding to ssDNA, the GGRQ motif in KpSSB will be dynamic and no longer bound by KpSSBn (Figure 10C). Our laboratory is currently attempting to obtain crystals of the KpSSB–ssDNA complex for this investigation.

Cases involved in self-binding to regulate its DNA-binding activity are found in many DNA-interaction proteins, for example, the initiation factor σ^70^, whose negative-charged subdomain 1.1 acts as a DNA mimic, which competes with promoter DNA for the binding site on domain 4 [89,90,91]. The negative-charged subdomain 1.1 in σ^70^ can regulate the binding ability to the promoter DNA during the RNA transcription initiation stage. Many DNA mimic proteins and peptides function by occupying the DNA binding sites of DNA binding proteins to prevent these sites from being accessed by DNA [92,93,94,95]. For SSB, SSB-Ct is probably in the case as a kind of DNA mimic [13]. Although the GGRQ motif in SSB does not have DNA-like negative surface charge distributions, this motif can compete with ssDNA for binding by the three ssDNA-binding residues, Asn14, Lys74, and Gln77 in SSBn. Due to pre-occupying the ssDNA binding sites, the GGRQ motif might also be considered to function as a kind of DNA mimic peptide.

The binding site on PriB for ssDNA has been proposed to overlap with the binding sites of PriA and DnaT [28]. This hypothesis can explain a mechanism for a dynamic primosome assembly process, in which ssDNA is handed off from one primosome protein to another as a repaired replication fork is reactivated [28]. If so, some regions in PriA and DnaT may, therefore, serve as a DNA mimic for competing with ssDNA for binding to PriB. Given that the complex structure of PriA-PriB and DnaT-PriB is not yet available, these putative binding sites have not been identified. In contrast, the evidence from the complexed structure of PriB–ssDNA [47] and the thermodynamic analysis [46] indicate that the PriB dimer behaves like a protein with half-site reactivity, where only one monomer of the dimer can engage in interactions with the DNA and the partner protein(s). Thus, it remains to be explored whether the binding site of PriB for ssDNA is necessary to overlap the binding sites of PriA and DnaT.

Recently, we showed complex structures of PaSSB in which all four OB folds do not simultaneously participate in the binding to ssDNA [6,7]. As with the case of PriB, ssDNA bound by PaSSB only occupies half of the binding sites of two OB folds rather than four OB folds through the ssDNA-binding mode (SSB)_3:1_ [6]. In many cases, OB folds can be broad ligand binders to both ssDNA and protein [10]. For the tumor suppressor BRCA2 [96], two OB folds bind to ssDNA, and a third OB fold is involved in protein–protein interactions. For RPA, two distinct binding modes can be involved, two OB folds and four OB folds, respectively [97]. It is possible that the empty OB fold in SSB is open to allow sliding, as described in single molecule experiments [98,99]. Whether the GGRQ motif, which may be associated with a binding cycle to ssDNA in the noninteracting OB fold(s) observed in our KpSSB structure, can, therefore, regulate the timing of ssDNA binding or sliding of SSB via reptation remains to be experimentally elucidated.

The number of OB-fold proteins has grown rapidly in recent years. The understanding of how they interact with ssDNA [100] and RNA [101] has improved considerably. The OB-fold structure is highly dynamic and supports a binding surface for protein–protein interaction and protein-nucleic acid binding. Loops linking β-strands can adopt different conformations to open or close the β-barrel. The nucleic acid-binding domains of many OB-fold proteins are similar in structure [10]. To execute the specific mission in physiology, these OB-fold proteins may contain additional different functional domains and regions, such as SSBc in SSB. The amino acid residues in SSBc are not conserved in amino acid residues identity and the gene product length, such as for SaSsbA [59], SaSsbB [58], and SaSsbC [57]. Unlike the Gram-negative bacterial SSBs [18,56], these Gram-positive bacterial SSBs did not stimulate the activity of PriA. It is possible that these differences are evolved gradually in each SSB to fit the precise need for binding to their different partner proteins, e.g., the different PriA-directed primosome assemblies [102]. The three PXXP motifs [1,16,17,55,86,87,103] in the IDL of EcSSB are known to mediate the different protein–protein interactions. Most Gram-negative bacterial SSBs, such as StSSB [51] and KpSSB [49], also contain these PXXP motifs, although with a minor modification [56]. In PaSSB, the second and third PXXP motifs are not significant [56]. This may be the reason that PaSSB could not enhance the activity of PriA [56].

In conclusion, we characterized and compared the ssDNA-binding properties of untagged KpPriB, KpSSB, and PriB-SSBc. Through protein chimeragenesis, the SSBc fused with KpPriB can significantly enhance the ssDNA-binding affinity, change the binding behavior, and further stimulate the PriA activity. SSBc did not mediate a dimeric PriB to be a tetramer, such as KpSSB. Our crystal structure revealed the dynamic movement of the GGRQ motif in SSBc as a part of the ssDNA-binding cycle in SSB. More complex structures of PriB are useful in improving our understanding of the primosome assembly mechanism(s).

## 4. Materials and Methods

### 4.1. Construction of Plasmids

Construction of the KpPriA [56], tag-free KpSSB [60] and tag-free KpPriB [31] expression plasmids has been reported. For PriB-SSBc, we constructed the plasmid following several steps. To obtain an additional cutting site (SacI) for fusing PriB and SSBc fragments, the pET21b-PriB [48] and pET21b-KpSSB (with the stop codon to avoid having a His tag fused with the gene product) [60] plasmids were mutated to create a desired SacI restriction site (aa 98–99 for pET21b-PriB/SacI and aa 111–112 for pET21b-SacI/KpSSB, respectively). The primers (GAGCAGATTGAGCTCATAGATTCTGGA and TCCAGAAT-CTATGAGCTCAATCTGCTC) were used for the E98E/L99L-engineered pET21b-KpPriB. The primers (GGCACCATGCAGGAGCTCGGCGGCCGT and ACGGCCGCCGAGCTC-CTGATGGTGCC) were used for the M111E/L112L-engineered pET21b-KpSSB. The E98E/L99L-engineered pET21b-PriB plasmid was cut with NdeI and SacI restriction enzymes, and the fragment KpPriB(1–98) was purified. Meanwhile, the M111E/L112L-engineered pET21b-KpSSB plasmid was also treated with NdeI and SacI restriction enzymes, and the resultant DNA fragment pET21b-KpSSB(112–174) was purified and then ligated with the insert KpPriB(1–98) DNA fragment. The resultant plasmid pET21b-KpPriB-KpSSBc will express KpPriB1–98 fused KpSSB112–174 (PriB-SSBc). Note that PriB-SSBc has 161 amino acid residues and does not have any artificial residues.

### 4.2. Protein Expression and Purification

Purification of the recombinant KpPriA [56], tag-free KpSSB [60] and tag-free KpPriB [31] has been reported. Briefly, KpSSB was purified by the precipitation of ammonia sulfate, Q, and Heparin column chromatographies. Unlike KpSSB, recombinant KpPriB and PriB-SSBc could be purified from the soluble supernatant only in a single chromatographic step using the SP column by the AKTA-FPLC system (GE Healthcare Bio-Sciences, Piscataway, NJ, USA). The recombinant tag-free PriB-SSBc was expressed and purified using the protocol described previously for KpPriB [31]. PriB-SSBc was expressed in *E. coli* BL21(DE3) cells with the expression vector by incubating with 1 mM isopropyl thiogalactopyranoside. The cells overexpressing the protein were resuspended in Buffer A (20 mM Tris–HCl and 100 mM NaCl, pH 5.9), and disrupted by sonication on ice. The soluble supernatant containing PriB-SSBc was applied to the SP column (GE Healthcare Bio-Sciences, Piscataway, NJ, USA). PriB-SSBc was eluted with a linear NaCl gradient from 0.1 to 1 M with Buffer A using the AKTA–FPLC system and dialyzed against Buffer B (20 mM HEPES and 100 mM NaCl, pH 7.0). The purity of these proteins was determined by Coomassie-stained SDS–PAGE (Mini-PROTEAN Tetra System; Bio-Rad, CA, USA).

### 4.3. Gel-Filtration Chromatography

Gel-filtration chromatography was carried out by the AKTA-FPLC system. In brief, purified PriB-SSBc (4 mg/mL) in Buffer B was applied to a Superdex 200 prep grade column (GE Healthcare Bio-Sciences, Piscataway, NJ, USA) equilibrated with the same buffer. The column was operated at a flow rate of 0.5 mL/min, and 0.5-mL fractions were collected. The proteins were detected by measuring the absorbance at 280 nm. The column was calibrated with proteins of known molecular weight: thyroglobulin (670 kDa), γ-globulin (158 kDa), ovalbumin (44 kDa), myoglobin (17 kDa), and vitamin B12 (1.35 kDa). The *K*_av_ values for the standard proteins and PriB-SSBc were calculated from the equation: *K*_av_ = (*V*_e_ − *V*_o_)/(*V*_c_ − *V*_o_), where *V*_o_ is column void volume, *V*_e_ is elution volume, and *V*_c_ is geometric column volume.

### 4.4. Preparation of dsDNA Substrate

The dsDNA substrate PS4/PS3-dT30 [57] was used for ATPase assay. PS4/PS3-dT30 was prepared at a 1:1 concentration ratio of PS4 and PS3-dT30. PS4/PS3-dT30 was formed in 20 mM HEPES (pH 7.0) and 100 mM NaCl by briefly heating at 95 ˚C for 5 min and by slowly cooling to room temperature overnight.

### 4.5. ATPase Assay

KpPriA ATPase assay was performed with 0.4 mM [γ^−32^P] ATP and 0.025 μM KpPriA in reaction buffer containing 40 mM Tris (pH 8.0), 10 mM NaCl, 2 mM DTT, 2.5 mM MgCl_2_, and 0.1 μM PS4/PS3-dT30 DNA substrate. To study the effect, KpSSB (10 μM), KpPriB (10 μM), or PriB-SSBc (10 μM) was added into the assay solution. Aliquots (5 μL) were taken and spotted onto a polyethyleneimine cellulose thin-layer chromatography plate, which was subsequently developed in 0.5 M formic acid and 0.25 M LiCl for 30 m. Reaction products were visualized by autoradiography and quantified with a phosphorimager (Typhoon 9410 Molecular Imager; GE Healthcare Bio-Sciences, Piscataway, NJ, USA).

### 4.6. Crystallography

Purified KpSSB was concentrated to 14 mg/mL for crystallization. Crystals were grown at room temperature by hanging drop vapor diffusion in 20% PEG 3350, 0.2 M magnesium acetate tetrahydrate, pH 6.5. The crystals reached full size in 9–12 days. Data were collected using an ADSC Quantum-315r CCD area detector at SPXF beamline BL13C1 at NSRRC (Taiwan). All data integration and scaling were carried out using HKL-2000 [104]. There were four KpSSB monomers per asymmetric unit. The crystal structure of KpSSB was solved at 2.3 Å resolution with the molecular replacement software Phaser-MR [105] using EcSSB as model (PDB entry 1EYG). A model was built and refined with PHENIX [106] and Coot [107]. The final structure was refined to an *R*-factor of 0.211 and an *R*_free_ of 0.258 (Table 1). Atomic coordinates and related structure factors have been deposited in the PDB with accession code 7F2N.

### 4.7. EMSA

EMSA was conducted in accordance with a previously described protocol for SSB [64]. In brief, ssDNA was radiolabeled with [γ-^32^P] ATP (6000 Ci mmol^−1^; PerkinElmer Life Sciences, Waltham, MA) and T4 polynucleotide kinase (Promega, Madison, WI, USA). The protein (0–5 μM for KpSSB; 0–5 μM for PriB-SSBc; and 0–50 μM for KpPriB) was incubated for 30 m at 25 °C with 1.7 nM DNA substrate in a total volume of 10 μL in 20 mM Tris–HCl (pH 8.0) and 100 mM NaCl. Aliquots (5 μL) were removed from each of the reaction solutions and added to 2 μL of gel-loading solution (0.25% bromophenol blue and 40% sucrose). The resulting samples were resolved on 8% native polyacrylamide gel at 4 °C in TBE buffer (89 mM Tris borate and 1 mM EDTA) for 1 h at 100 V and visualized through phosphorimaging. A phosphor storage plate was scanned, and data regarding complex and free DNA bands were digitized for quantitative analysis. The ssDNA binding ability of the protein was estimated through linear interpolation from the concentration of the protein that bound 50% of the input DNA.

## Figures and Tables

**Table 1:** Data collection and refinement statistics.

Data Collection	
Crystal	KpSSB
Wavelength (Å)	1
Resolution (Å)	27.7–2.35
Space group	C_1_2_1_
Cell dimension *a*, *b*, *c* (Å) *β* (°)	108.39, 57.01, 93.80 103.72
Redundancy	3.4 (2.9)
Completeness (%)	97.7 (89.9)
<I/σI>	17.2 (3.0)
CC_1/2_	0.984 (0.915)
Refinement	
No. reflections	22807
*R*_work_/*R*_free_	0.211/0.258
No. atoms	
Protein	429
Water	120
r.m.s deviations	
Bond lengths (Å)	0.010
Bond angles (°)	1.31
Ramachandran plot	
Favored (%)	98.53
Allowed (%)	1.47
Outliers (%)	0
PDB entry	7F2N

Values in parentheses are for the highest resolution shell. CC_1/2_ is the percentage of correlation between intensities of random half-data sets.

**Table 2:** The formation of hydrogen bonds and salt bridges at the dimer–dimer interface of KpSSB and the corresponding residues in KpPriB.

KpSSB	Distance [Å]	Corr. Residues in KpPriB
L113(B)–Q77(C)	2.86	I100/A71
G5(B)–Q111(D)	2.92	None/E98
K8(B)–E81(D)	3.83	R4/R75
E81(B)–K8(D)	3.51	R75/R4
G114(A)–Q77(D)	2.92	D101/A71
R4(A)–Q111(C)	3.10	None/E98
R4(A)–M112(C)	3.73	None/L99
G5(A)–Q111(C)	2.82	None/E98
Q111(A)–G5(C)	2.52	E98/None
K8(A)–E81(C)	3.03	R4/ R75
E81(A)–K8(C)	3.62	R75/R4

The formation of hydrogen bonds and salt bridges at the dimer–dimer interface of KpSSB was analyzed by using PISA (Protein Interfaces, Surfaces and Assemblies), which is an automatic analytical tool for macromolecular assemblies in the crystalline state.

**Table 3:** ssDNA binding properties of KpSSB, KpPriB, and PriB-SSBc as analyzed by EMSA.

DNA	Protein	[Protein]_50_ (μM)	Complex Number
dT15	PriB-SSBc	ND	0
	KpPriB	ND	0
	KpSSB	ND	0
dT20	PriB-SSBc	2.12 ± 0.18	1
	KpPriB	ND	0
	KpSSB	ND	0
dT25	PriB-SSBc	1.15 ± 0.12	1
	KpPriB	14.1 ± 1.0	1
	KpSSB	0.58 ± 0.05	1
dT30	PriB-SSBc	0.94 ± 0.08	1
	KpPriB	9.2 ± 0.8	1
	KpSSB	0.55 ± 0.04	1
dT40	PriB-SSBc	0.84 ± 0.06	1
	KpPriB	9.1 ± 0.8	1
	KpSSB	0.23 ± 0.01	1
dT50	PriB-SSBc	0.29 ± 0.02	1
	KpPriB	5.6 ± 0.3	1
	KpSSB	0.17 ± 0.02	1
dT55	PriB-SSBc	0.33 ± 0.03	2
	KpPriB	4.9 ± 0.5	1
	KpSSB	0.13 ± 0.01	2
dT60	PriB-SSBc	0.23 ± 0.02	2
	KpPriB	4.8 ± 0.5	1
	KpSSB	0.05 ± 0.01	2

[Protein]_50_ was calculated from the titration curves of EMSA by determining the concentration of the protein (μM) needed to achieve the midpoint value for input ssDNA binding. For some oligonucleotides, input ssDNA binding was the sum of the intensities from the two separate ssDNA-protein complexes. Errors are standard deviations determined by three independent titration experiments.

**Table 4:** The formation of hydrogen bonds at the GGRQ motif in KpSSB.

Hydrogen Bond	Dist. [Å]
G114(B)–Q77(B)	3.9
G115(B)–Q77(B)	3.3
R116(B)–K74(B)	3.6
Q117(B)–N14(B)	3.4
G114(A)–Q77(D)	2.9
G114(C)–Q77(C)	3.3

The 114–GGRQ–117 motif-interacting residues N14, K74, and Q77 in KpSSBn are conserved in EcSSB (N14, K74, and Q77) and PaSSB (N13, K73, and Q76).

## Data Availability

Atomic coordinates and related structure factors were deposited in the PDB with accession code 7F2N.
